# Postnatal Growth Patterns and Deviations in Singleton and Twin Infants: A Prospective Cohort Study on Infants Appropriate and Small for Gestational Age

**DOI:** 10.1002/pdi3.70019

**Published:** 2025-11-30

**Authors:** Yannan Wan, Ting Pan, Zhanzhan Zhang, Yuanfeng Zhong, Qian Chen, Xuelin Xia, Lin Zhu, Li Chen, Xuan Zhang

**Affiliations:** ^1^ Growth, Development and Mental Health Center of Children and Adolescents Chongqing Key Laboratory of Child Neurodevelopment and Cognitive Disorders National Clinical Research Center for Child Health and Disorders Ministry of Education Key Laboratory of Child Development and Disorders Children's Hospital of Chongqing Medical University Chongqing China; ^2^ Guizhou Branch of Shanghai Children's Medical Center Shanghai Jiaotong University School of Medicine Guiyang China

**Keywords:** appropriate for gestational age, catch‐up growth, physical growth deviation, small for gestational age, twins

## Abstract

Small for gestational age (SGA) and twin infants are at increased risk of growth deviations, but postnatal catch‐up growth (CUG) patterns across singleton, twin, appropriate for gestational age (AGA), and SGA groups remain unclear. This prospective cohort study (*n* = 340) investigated the postnatal growth trajectories and physical growth deviations in singleton and twin AGA and SGA infants during the first year of life. The primary findings indicated that SGA infants exhibited rapid CUG in weight and head circumference within the first 6 months, whereas length catch‐up required a longer period. Twin SGA infants displayed distinct patterns: faster weight CUG but slower length CUG compared with singleton SGA infants, with gender differences—male SGA twins had lower length *z*‐scores than SGA singletons. The rate of deviation in physical growth of SGA infants improved significantly within the first year of life, with wasting being infrequent. In conclusion, the mechanism underlying SGA is more complex among twins than singletons. Twin SGA infants require prolonged growth monitoring, and regular follow‐up is essential to optimize growth outcomes and mitigate long‐term risks. This preliminary study offers a foundation for further investigation into the biological and environmental factors driving these differences.

## Graphical abstract

This is a cohort study including 340 infants stratified into small or appropriate for gestational age. We examined their early physical growth characteristics and described the differences in etiology and catch‐up growth patterns between singleton and twin small for gestational age infants.

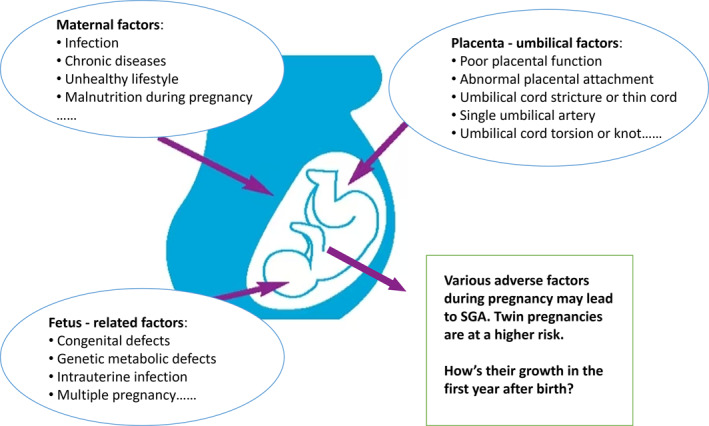

## 1 Introduction

Recent advancements in assisted reproductive technology (ART), perinatal care, and neonatal intensive care have contributed to a steady improvement in the survival rates of high‐risk infants. Consequently, the proportion of high‐risk infants who survive has increased. In China, the incidence of high‐risk infants ranges from 10% to 20% [1], making it a growing public health challenge. The global incidence of small for gestational age (SGA) infants ranged from 3% to 10% [2]. In China, the overall incidence was 6.6%, with a significantly higher rate observed among preterm infants (13.1%) compared to term infants (6.1%) [3]. These findings highlight notable regional and gestational‐related variations in SGA incidence. Because of factors such as placental resource competition and the unique physiological adaptations associated with multiple pregnancies, the incidence of SGA is significantly higher in twin pregnancies compared to singleton pregnancies. Globally, the incidence of SGA in twins ranged from 4.8% to 18.8%. In low‐income regions (such as sub‐Saharan Africa), the incidence was elevated due to maternal undernutrition and limited access to prenatal care. In China, the reported incidence of SGA in twins was notably high, ranging from 47.0% to 57.4% [4]. According to data from the Chinese Maternal and Child Health Association in 2019, the incidence of twin pregnancy was 3.7%. It is worth noting that approximately 32% of these twin pregnancies resulted from ART, a factor that significantly increases the risk of SGA in twins [5]. Moreover, the estimated neonatal mortality rate for SGA twins was significantly higher compared with that for appropriate for gestational age (AGA) twins (53 per 1000 live births compared to 8 per 1000, respectively) [6]. Twin SGA infants are at elevated risk for complications, including fetal distress and neonatal asphyxia, contributing to increased utilization of Neonatal Intensive Care Unit (NICU) services. The demand for intensive neonatal care among SGA infants varied considerably across regions. For instance, the NICU utilization rate for SGA infants was 42.4% in the Middle East and North Africa, 6.5% in Southeast Asia, and approximately 5.9% in China (comparable to rates in Nordic countries) [7]. These figures represent significant regional disparities in healthcare resource allocation and neonatal care capacity.

High‐risk infants are more susceptible to delays in physical growth, neuropsychological development, or both, as well as nutritional deficiencies [8]. After birth, most SGA and twin infants experience catch‐up growth (CUG) to achieve the expected level of physical development. Retrospective studies conducted by our research group have indicated potential differences in postnatal CUG patterns between twins and singletons [7]. To clarify these variations, it is imperative to conduct prospective cohort studies to examine physical growth trajectories, differences in CUG, and deviations in physical growth among SGA and AGA singletons and twins during the first year of life.

Hence, this study aims to explore variations in growth patterns during the critical period of rapid postnatal development, focusing on the incidence of CUG and growth deviations between AGA and SGA singletons and twins within the first year of life. The findings are expected to guide the development of a comprehensive management approach for infants with varying birth statuses in the first year of life.

## 2 Objects and Methods

### 2.1 Objects

This prospective cohort study was conducted in the Department of Child Health Care, Children's Hospital of Chongqing Medical University, involving the enrollment of infants < 3 months old from October 2021 to March 2022.

The inclusion criteria were as follows: Singleton and twin infants exhibiting a birth weight below the 90th percentile; a gestational age between ≥ 28 weeks and < 42 weeks; and the absence of severe medical conditions such as congenital heart disease, congenital anomalies, inherited metabolic diseases, or gastrointestinal disorders that could significantly affect feeding. Long‐term residency with Chongqing household registration, a signed informed consent, and voluntary agreement to participate in the follow‐up from the guardians were also required to be eligible.

The exclusion criteria were as follows: Infants who were hospitalized for over 30 days during the neonatal period, underwent a major surgical procedure, or had severe neurological conditions, such as encephalitis, epilepsy, severe craniocerebral trauma, or brain tumors. Infants who experienced severe cardiac, hepatic, renal, respiratory, or gastrointestinal disorders during follow‐up or declined to participate in the follow‐up were also excluded.

Recruitment drives were conducted through hospital and departmental WeChat posts and in‐clinic promotion. The posts included details about the study aims, eligibility criteria, potential benefits, and contact numbers. Clinicians explained the study to eligible parents, followed by signing informed consent after agreement, either in person at the department or via immediate contact with researchers. Physical measurements (weight, length, and head circumference) along with demographic data (sex, gestational age, mode of delivery, birth weight, parental height, parental education, and recent illnesses) were collected up to 1 year of life.

### 2.2 Research Methods

#### 2.2.1 Data Cleansing

To preserve the privacy of study participants, unique identifications (IDs) were generated by matching patient details such as name, consultation ID number, parental information, date of birth, and home phone number. Data desensitization measures were implemented after data analysis to protect participant privacy and ensure data confidentiality.

#### 2.2.2 Physical Measurements

Two nurses with over 5 years of experience in physical measurements and specialized training from the Department of Pediatric Health Care at Children's Hospital of Chongqing Medical University were recruited as measurers to ensure the accuracy of physical index measurements. Standardized and validated measurement tools were employed throughout the data collection process. Weight was measured using a seated disk‐type lever scale with a precision of 0.05 kg. Length was measured using a wooden infant measuring bed, equipped with a fixed headboard and a movable footboard, featuring rulers on both sides with an accuracy of 0.1 cm. The infant was positioned supine along the midline of the measuring bed. Head circumferences were measured using a nonstretchable soft measuring tape ruler with a precision of 0.1 cm. The tape was carefully positioned to traverse standardized anatomical landmarks, specifically the superior border of the superciliary arch anteriorly and the external occipital protuberance posteriorly.

#### 2.2.3 Grouping and Evaluation Standards for Physical Growth Indicators

According to the 2015 Chinese birth weight guidelines [9], newborns were categorized as preterm (if born before 37 weeks of gestation) and term (if born between 37 and 42 weeks of gestation). Singletons and twin infants were categorized into AGA and SGA groups. Infants with birth weights between the 10th and 90th percentiles for their gestational age and sex were classified as AGA, whereas those with birth weights below the 10th percentile were classified as SGA.

Standard deviation score (SDS) values (*z*‐scores) for birth weights, as well as for weight, length, head circumference, and body proportionality at each follow‐up visit were computed using the World Health Organization's Anthro software (version 3.2.2, 2011). These computed *z‐*scores included weight‐for‐age (WAZ), length‐for‐age (LAZ), head circumference‐for‐age (HCZ), and weight‐for‐length (WFLZ). The *z*‐scores were computed using monthly age intervals to ensure age precision. For preterm infants, age was corrected for prematurity before *z*‐score calculation.

The definitions of CUG and failed CUG in SGA infants vary across studies. Some define CUG as an increment in the SDS of ≥ 0.67 [10]. In contrast, others defined it based on the achievement of a particular growth level of specific indicators (such as weight or length reaching the 10th percentile for age and gender [11], or height exceeding ‐2 SDS [12]). Failed CUG is typically defined as a height lower than ‐2 SDS at 2–4 years of age. Since SGA infants with failed CUG often require clinical intervention, a *z*‐score threshold of > −2 is considered a practical and clinically relevant criterion. Accordingly, LAZ > −2 was utilized as the benchmark for CUG in this study. Deviations in physical growth were defined as follows: WAZ < −2, LAZ < −2, WFLZ < −2, and WFLZ > 2.

The pediatric healthcare follow‐up plan consisted of monthly visits for infants aged 0–6 months and visits every two to three months for those aged 6–12 months. However, adherence to the planned schedule was not always feasible due to the COVID‐19 pandemic and associated residential lockdowns during the follow‐up period. To analyze the intergroup differences in physical growth *z*‐scores and the rate of deviation in physical growth, we used 3, 6, 9, and 12 months of age as time points. When multiple visits occurred within a given time point, the average of the relevant measurements was used for analysis. Additionally, physical growth data from all follow‐ups (including those outside the designated time points) were used to construct fitted growth curves, thereby maximizing data usage to visualize growth trends across different groups.

### 2.3 Statistical Analysis

Statistical analyses were conducted using SPSS software (version 26.0). The distribution of the *z*‐scores of physical indicators in each group was examined using a Shapiro–Wilk normality test. Data that conformed to a normal distribution is presented in the form of the mean value and the standard deviation (SD), whereas data that did not conform to a normal distribution were expressed as the median and the interquartile range (IQR). Generalized estimating equations (GEEs) were used to evaluate the differences in the *z*‐scores of physical growth indicators among groups. Comparisons for the deviation in the prevalence rates of physical growth were conducted using the chi‐square test. A *p*‐value < 0.05 was considered to be statistically significant. All the charts were generated using Origin Pro 2021. Several common curve‐fitting functions were evaluated based on their coefficient of determination (*R* ^2^) to model growth trajectories. The logistic and Gompertz functions, which demonstrated the highest *R* ^2^ values (closest to 1), were selected for fitting the growth curves.

## 3 Results

### 3.1 General Information

A total of 340 cases of singletons and twin infants up to one year of age were included in this study, culminating in total of 2222 follow‐up appointments. Of these infants, 97 were cases of AGA (28.5%) and 106 were cases of SGA (31.2%) in singleton pregnancies; and 93 were cases of AGA (27.4%) and 44 were cases of SGA (12.9%) in twin pregnancies. There was no significant difference in sex distribution between the groups (singletons: *p* = 0.90, twins: *p* = 0.06). Further details are presented in Table 1.

**TABLE 1:** The general characteristics of AGA and SGA in singletons and twins.

Group		Singleton		Twins	
		AGA (*n* = 97)	SGA (*n* = 106)	AGA (*n* = 93)	SGA (*n* = 44)
Sex	Male (*n*, [%])	53 (26.1)	57 (28.1)	54 (39.4)	18 (13.1)
	Female (*n*, [%])	44 (21.7)	49 (24.1)	39 (28.5)	26 (19.0)
*z*‐score of birth weight (kg; mean ± SD)	Full‐term	−0.07 ± 0.68^a^ ^,^ ^c^	−1.73 ± 0.57^d^	−1.05 ± 0.52^b^	−2.44 ± 0.55
	Preterm	−2.46 ± 1.17^a^	−3.14 ± 0.78	−1.98 ± 0.91^b^	−3.52 ± 0.63
Preterm birth rate (*n*, [%])		37 (38.1)^a^ ^,^ ^c^	13 (12.3)	52 (55.9)^b^	11 (25.0)
Gestational age (weeks; mean ± SD)		37.5 ± 2.6^a^ ^,^ ^c^	38.5 ± 1.4	36.3 ± 1.4^b^	36.9 ± 1.1
Intensive feeding rate (*n*, [%])		12 (12.4)	12 (11.3)	12 (12.9)	7 (15.9)

*Note:* The superscripts a–d indicate the statistically significant differences between the two groups (*p* < 0.05).

Abbreviations: AGA, appropriate for gestational age; SD, standard deviation; SGA, small for gestational age.

^a^ Singleton AGA versus Singleton SGA.

^b^ Twin AGA versus Twin SGA.

^c^ Singleton AGA versus Twin AGA.

^d^ Singleton SGA versus Twin SGA.

Among full‐term infants, the mean birth weight (BW) of singleton AGA infants was significantly higher compared to that of twin AGA (*p* < 0.01), and the mean BW of singleton SGA was significantly higher compared to that of twin SGA (*p* < 0.01). It is worth noting that for preterm infants, no significant differences were observed between singleton AGA and twin AGA infants (*p* = 0.21) and between singleton SGA and twin SGA (*p* = 0.92). The mean BW of preterm singleton and twin AGA infants was determined to be close to the median based on the Fenton preterm growth chart.

The mean gestational age of singletons was slightly higher compared to that of twins (*p* < 0.01). There were significant differences in the preterm birth rates among the groups, but no significant difference was detected in the intensive feeding rates (including the use of breast milk fortifiers and preterm formula) among the groups (singletons: *p* = 0.82, twins: *p* = 0.63).

### 3.2 Physical Growth and Growth Deviation of Singleton and Twin AGA and SGA Infants in the First Year of Life

#### 3.2.1 Differences in the Physical Growth Levels and Body Proportions Between Singletons and Twin AGA and SGA Infants in the First Year of Life

The generalized estimating equations (GEEs) model was used to analyze the dynamic changes in the WFLZ, LAZ, WAZ, and HCZ of singleton AGA, singleton SGA, twin AGA, and twin SGA infants at the ages of 0–3 months, 3–6 months, 6–9 months, and 9–12 months. Table 2a shows the *z*‐scores in all groups. Table 2b shows the results of the GEE analysis for WFLZ, LAZ, WAZ, and HCZ.

**TABLE 2a:** WAZ, LAZ, HCZ, and WFLZ of AGA and SGA in twins and singletons under 1 year of age.

Group and age in months (m)	Singleton AGA	Singleton SGA	Twin AGA	Twin SGA
	Mean ± SD/median (IQR)	Mean ± SD/median (IQR)	Mean ± SD/median (IQR)	Mean ± SD/median (IQR)
WFLZ				
0–3	0.37 ± 0.84	0.14 ± 0.93	0.34 ± 0.99	0.16 ± 0.89
3–6	0.63 (0.00, 1.13)	−0.17 (−0.66, 0.67)	0.50 (−0.08, 1.11)	0.19 (−0.36, 0.55)
6–9	0.49 (−0.01, 1.04)	0.12 (−0.72, 0.45)	0.36 (−0.17, 1.13)	0.10 (−0.58, 0.48)
9–12	0.27 (−0.42, 0.95)	−0.21 (−1.09, 0.29)	0.58 (−0.19, 1.01)	0.11 (−0.77, 0.44)
WAZ				
0–3	0.30 (−0.39, 0.80)	−1.46 (−1.89, −0.74)	0.05 (−0.67, 0.78)	−1.25 (−1.85, −0.53)
3–6	0.28 (−0.24, 1.00)	−0.90 (−1.51, −0.44)	0.03 (−0.40, 0.90)	−0.96 (−1.49, −0.31)
6–9	0.27 (−0.26, 0.87)	−0.98 (−1.50, −0.31)	0.33 (−0.36, 0.90)	−0.73 (−1.63, −0.05)
9–12	0.17 (−0.42, 0.75)	−0.71 (−1.41, −0.33)	0.31 (−0.36, 0.86)	−0.81 (−1.44, −0.08)
LAZ				
0–3	−0.93 ± 1.42	−1.77 ± 0.94	−1.38 ± 0.92	−1.92 ± 0.69
3–6	−0.07 (−0.69, 0.47)	−1.14 (−1.73, −0.75)	−0.44 (−0.46, 0.56)	−1.34 (−1.87, −0.94)
6–9	−0.36 ± 0.97	−1.24 ± 0.84	−0.29 ± 0.90	−1.24 ± 0.95
9–12	−0.09 (−0.95, 0.74)	−1.07 (−1.68, −0.65)	0.12 (−0.81, 0.74)	−1.07 (−1.64, −0.50)
HCZ				
0–3	−0.58 ± 1.37	−1.12 ± 0.98	−0.97 ± 0.93	−1.16 ± 0.71
3–6	0.15 (−0.58, 0.65)	−0.87 (−1.34, −0.27)	0.14 (−0.68, 0.39)	−0.62 (−1.31, −0.10)
6–9	−0.10 (−0.60, 0.53)	−0.73 (−1.39, −0.17)	−0.13 (−0.57, 0.44)	−0.60 (−1.39, 0.07)
9–12	0.07 (−0.57, 0.68)	−0.69 (−1.28, −0.06)	0.19 (−0.77, 0.55)	−0.35 (−1.08, 0.16)

Abbreviations: AGA, appropriate for gestational age; HCZ, *z*‐score of head circumference‐for‐age; IQR, interquartile range; LAZ, *z*‐score of length‐for‐age; SD, standard deviation; SGA, small for gestational age; WAZ, *z*‐score of weight‐for‐age; WFLZ, *z*‐score of weight‐for‐length.

**TABLE 2b:** Analysis of WAZ, LAZ, HCZ, and WFLZ of AGA and SGA in twins and singletons by GEE analysis.

Indicator	Group	*β*	95% CI	*p*
WFLZ	Singleton AGA	Ref.		
	Singleton SGA	−0.54	−0.77, −0.312	< 0.01
	Twin AGA	0.05	−0.195, 0.288	0.71
	Twin SGA	−0.38	−0.655, −0.104	0.01
	0–3 months	Ref.		
	3–6 months	0.00	−0.086, 0.09	0.97
	6–9 months	−0.09	−0.199, 0.028	0.14
	9–12 months	−0.31	−0.448, −0.167	< 0.01
LAZ	Singleton AGA	Ref.		
	Singleton SGA	−1.22	−1.432, −1	< 0.01
	Twin AGA	0.06	−0.185, 0.299	0.65
	Twin SGA	−1.18	−1.474, −0.886	< 0.01
	0–3 months	Ref.		
	3–6 months	0.18	0.115, 0.246	< 0.01
	6–9 months	0.32	0.229, 0.405	< 0.01
	9–12 months	0.31	0.194, 0.418	< 0.01
WAZ	Singleton AGA	Ref.		
	Singleton SGA	−1.23	−1.455, −1.002	< 0.01
	Twin AGA	0.06	−0.184, 0.296	0.65
	Twin SGA	−1.10	−1.391, −0.815	< 0.01
	0–3 months	Ref.		
	3–6 months	0.24	0.16, 0.313	< 0.01
	6–9 months	0.31	0.203, 0.412	< 0.01
	9–12 months	0.22	0.088, 0.355	< 0.01
HCZ	Singleton AGA	Ref.		
	Singleton SGA	−0.84	−1.06, −0.61	< 0.01
	Twin AGA	−0.05	−0.282, 0.177	0.65
	Twin SGA	−0.72	−0.987, −0.444	< 0.01
	0–3 months	Ref.		
	3–6 months	0.02	−0.046, 0.088	0.54
	6–9 months	0.11	0.028, 0.195	0.01
	9–12 months	0.13	0.021, 0.236	0.02

Abbreviations: *β*, regression coefficient; AGA, appropriate for gestational age; GEE, generalized estimating equation; HCZ, *z*‐score of head circumference‐for‐age; LAZ, *z*‐score of length‐for‐age; Ref., this group serves as the reference group; SGA, small for gestational age; WAZ, *z*‐score of weight‐for‐age; WFLZ, *z*‐score of weight‐for‐length.

**FIGURE 1:**
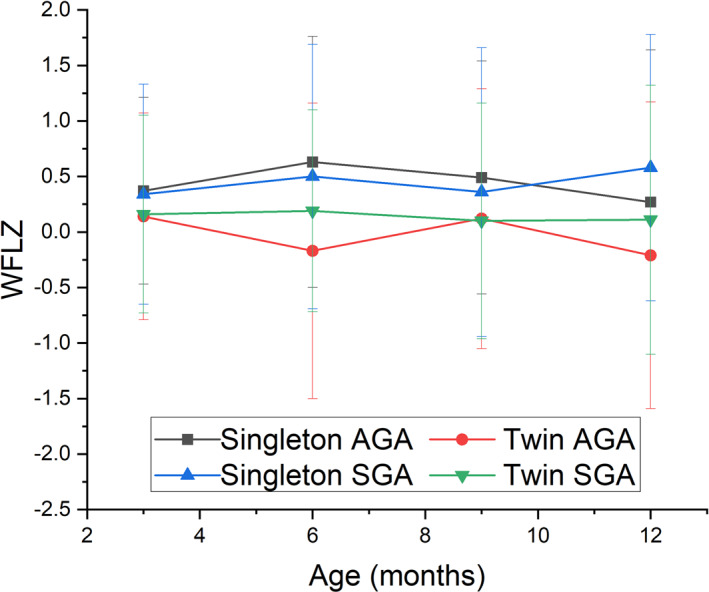
The WFLZ of SGA and AGA in twins and singletons under 1 year of age. AGA, appropriate for gestational age; SGA, small for gestational age; WFLZ, *z*‐score of weight‐for‐length.

In terms of body proportion, as shown in Table 2b, there were significant differences in WFLZ among groups. Further pairwise comparisons revealed that the WFLZ of AGA infants was significantly higher than that of SGA infants, in both singletons and twins. However, although the WFLZ of SGA was significantly lower than that of AGA, its *z*‐scores were approximately 0 (95% CI: −0.21 to 0.19), indicating that the average body proportion of SGA infants was at a normal level. We attempted to generate fitted curves for WFL across different groups; however, the scatter plots revealed substantial dispersion among individual data points. As a result, the *R* ^2^ was notably low, indicating poor model fit and limited suitability of curve fitting for weight‐for‐length (WFL) in this dataset. This indicates substantial individual differences in body fatness and thinness among these groups of infants. Although there were statistically significant intergroup differences in the WFLZ between AGA and SGA, the mean values of WFLZ across the four groups slightly fluctuated, with a significant concentration at a moderate level, indicating that the overall body proportion was normal (Figure 1).

In terms of the growth level, Table 2a showed that from 0 to 6 months of age, the *z*‐scores of WFLZ, WAZ, LAZ, and HAZ increased significantly, indicating that the CUG was the fastest at this stage. The *z*‐scores of length, weight, and head circumference of singleton SGA increased by 0.56, 0.58, and 0.54, respectively, whereas those of twin SGA increased by 0.29, 0.63, and 0.25, which suggests that the weight catch‐up of twin SGA was faster than that of singleton SGA, whereas the catch‐ups of length and head circumference of singleton SGA were faster than that of the twin SGA. After 6 months of age, the *z*‐scores of various indicators generally showed a trend that was still increasing but slower than before. The GEE model was used to analyze the dynamic changes in LAZ, WAZ, and HCZ among the four groups. The results, as shown in Table 2b, indicated significant differences in these three physical growth indicators among the four groups of infants. Further pairwise comparisons showed that the *z*‐scores of these indicators in singleton AGA were significantly higher than those in singleton SGA, and the *z*‐scores in twin AGA were significantly higher than those in twin SGA. However, there were no significant differences between singleton AGA and twin AGA, as well as between singleton SGA and twin SGA.

**FIGURE 2:**
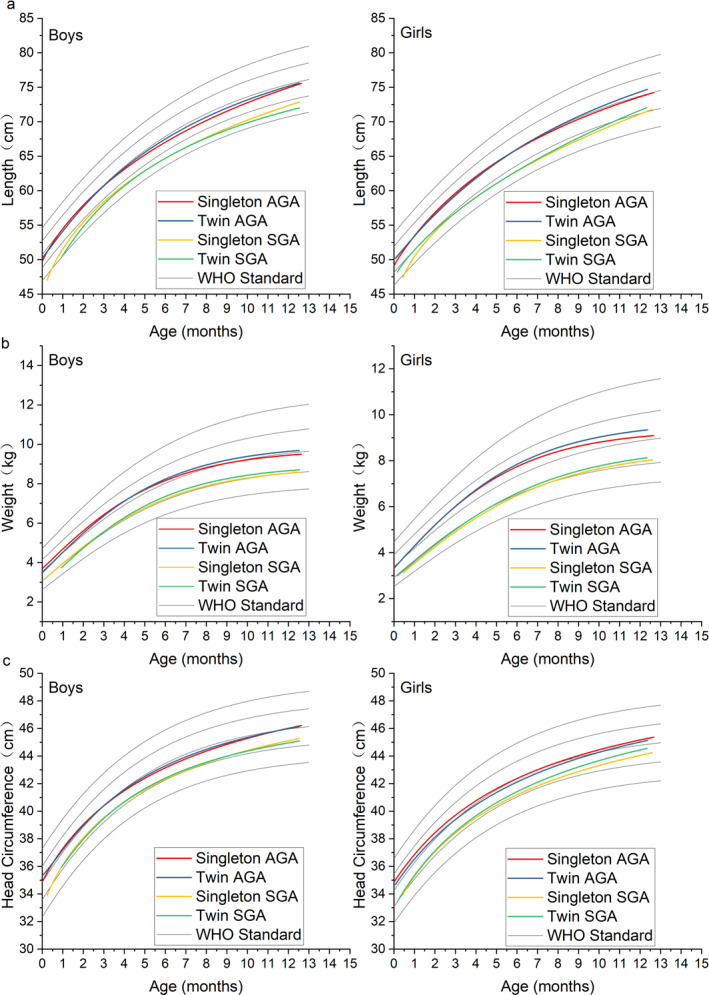
Fitted growth chart of length (2a), weight (2b) and head circumference (2c) within the first year after birth. AGA, appropriate for gestational age; SGA, small for gestational age; WHO, World Health Organization.

To elucidate the growth trends of infants in each group, the fitted curves were constructed using appropriate functions leveraging insights from the scatter plots on the physical examination data of the four groups of children at each follow‐up (Figure 2a–c). The fitted curves exhibited a superior *R* ^2^ value of 0.75, indicating an ideal fitting effect (the detailed fitting information is provided in the Supporting Information S1). For reference, each graph simultaneously displays the five World Health Organization (WHO) standard *z*‐score curves, corresponding to *z* = −2, −1, 0, 1, and 2, arranged sequentially from lowest to highest, to facilitate visual comparison with normative growth benchmarks.

The fitted growth curves of both singleton and twin AGA infants resemble the WHO *z* = 0 curve (Figure 2a). The length of male SGA twins was lower compared to that of SGA singletons during the initial stages, with leveling observed at approximately 3 months of age. However, the growth rate in length of the male SGA twins gradually decreased to lower levels compared to that of the SGA singletons after 9 months of age. In contrast, the length of female SGA twins was higher than that of SGA singletons at the initial stage; however, the length of female SGA singletons rapidly levels, with the rates for both groups remaining comparable after 2 months of age. From the growth curve, the length of SGA males ranged from −2SD to −1SD at 1 year of age, whereas that of females ranged from −1SD to 0SD.

Initially, the weight of male twin appropriate for gestational age (AGA) infants was slightly lower than that of singleton AGA infants, and the weight of male twin small for gestational age (SGA) infants was slightly lower than that of singleton SGA infants. However, this difference had disappeared below 3 months of age, with the growth lag of twins relative to singletons having been caught up. It is worth noting that after 6 months of age, the weight of twins was slightly higher than that of singletons, especially for AGA. On the fitted curves, the weight levels of both male and female SGA infants at 12 months of age were observed to be at or slightly above the −1SD line.

The growth trend of the head circumference of AGA infants resembles the WHO *z* = 0 curve (Figure 2c). It is worth noting that SGA exhibited a slightly higher CUG, which was pronounced in females, than AGA. The head circumference of SGA at 1 year of age ranged from −1SD to 0SD.

#### 3.2.2 Deviations in Growth Within the First Year of Life Between Singleton and Twin Infants

**FIGURE 3:**
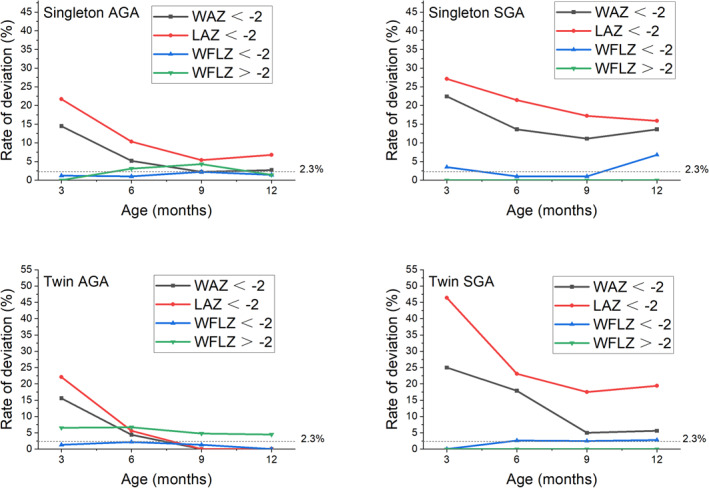
The deviation of physical growth indicators among singleton and twin AGA and SGA infants at different months. AGA, appropriate for gestational age; LAZ, *z*‐score of length‐for‐age; SGA, small for gestational age; WAZ, *z*‐score of weight‐for‐age; WFLZ, *z*‐score of weight‐for‐length.

Based on the definitions and guidelines set forth by WHO, we determined the prevalence of WAZ < −2SD (underweight), LAZ < −2SD (stunting), WFLZ < −2SD (wasting) and WFLZ > 2SD (overweight), and analyzed the differences among the groups. The details are presented in Table 3 and Figure 3. It is worth noting that approximately 2.3% of individuals typically exhibit a *z*‐score lower than −2SD, which is an inherent characteristic of the normal curve and not indicative of health problems. Consequently, physical growth deviations in infants are not an absolute indication of an underlying health problem. Accordingly, a reference line at 2.3% was added in Figure 3, indicating that the health risk may increase when the prevalence of deviation exceeds this value.

**TABLE 3:** The deviation of physical growth indicators among singleton, twins AGA, and SGA at different months (*n*, [%]).

Indicators	Age (month)	Singleton AGA (*n*, [%])	Singleton SGA (*n*, [%])	Twins AGA (*n*, [%])	Twins SGA (*n*, [%])	*p*
WAZ < −2	Birth	23^a^ (23.7)	37 (34.9)	22^b^ (23.7)	37 (84.1)	< 0.01
	0–3	12 (14.5)	19 (22.4)	12 (15.6)	7 (25.0)	0.40
	3–6	5^a^ (5.2)	14 (13.6)	4^b^ (4.4)	7 (17.9)	0.02
	6–9	2^a^ (2.2)	11 (11.1)	0^b^ (0.0)	6 (15.0)	< 0.01
	9–12	2^a^ (2.7)	12 (13.6)	0 (0.0)	2 (5.6)	< 0.01
LAZ < −2	0–3	18 (21.7)	23 (27.1)	17 (22.1)	13 (46.4)	0.06
	3–6	10^a^ (10.3)	22 (21.4)	5^b^ (5.6)	9 (23.1)	< 0.01
	6–9	5^a^ (5.4)	17 (17.2)	0^b^ (0.0)	7 (17.5)	< 0.01
	9–12	5^a^ (6.8)	14 (15.9)	0^b^ (0.0)	7 (19.4)	< 0.01
WFLZ < −2	0–3	1 (1.2)	3 (3.5)	1 (1.3)	0 (0.0)	0.73
	3–6	1 (1.0)	1 (1.0)	2 (2.2)	1 (2.6)	0.69
	6–9	2 (2.2)	1 (1.0)	1 (1.2)	1 (2.5)	0.75
	9–12	1 (1.4)	6 (6.8)	0 (0.0)	1 (2.8)	0.07
WFLZ > 2	0–3	0^c^ (0.0)	0 (0.0)	5 (6.5)	0 (0.0)	0.01
	3–6	3^c^ (3.1)	0 (0.0)	6 (6.7)	0 (0.0)	0.02
	6–9	4 (4.3)	0 (0.0)	4 (4.8)	0 (0.0)	0.07
	9–12	1 (1.4)	0 (0.0)	3 (4.5)	0 (0.0)	0.11

*Note:* The superscripts a–c indicates the statistically significant differences between the two groups (*p* < 0.05). Due to the COVID‐19 pandemic during the follow‐up period, a small number of children failed to attend the follow‐up at the scheduled time‐point. The percentages in this table were calculated based on the actual number of children who visited on time. For example, there were 97 singleton AGA children in total, 83 of whom completed the 3‐month‐old follow‐up. Among these 83 children, 12 had a WAZ below −2, so the percentage was 12/83 (14.5%).

Abbreviations: AGA, appropriate for gestational age; LAZ, *z*‐score of length‐for‐age; SGA, small for gestational age; WAZ, *z*‐score of weight‐for‐age; WFLZ, *z*‐score of weight‐for‐length.

^a^ Singleton AGA versus Singleton SGA.

^b^ Twin AGA versus Twin SGA.

^c^ Singleton AGA versus Twin AGA.

At the majority of follow‐up time points, the prevalence of stunting and underweight were significantly higher in the SGA group compared to the AGA group. The prevalence of stunting showed a downward trend before the age of 9 months in all groups. During this period, the singleton SGA group maintained a relatively rapid downward trend, whereas the downward trends of the AGA group and the twin SGA group declined during 6–9 months. However, after 9 months, the prevalence of stunting in the twin SGA group slightly increased. The prevalence of underweight in all groups showed a significant downward trend before the age of 9 months, with the fastest decline occurring within the first 6 months. However, the prevalence of underweight stabilized after 9 months. The downward trend of the underweight prevalence in SGA twins within 6 months was more pronounced than in SGA singletons, suggesting that SGA twins had a faster rate of early weight CUG. The prevalence of stunting in the SGA group consistently exceeded that of underweight throughout the first year of life, in both singletons and twins.

Conversely, the twin AGA group exhibited a higher prevalence of overweight than the singleton AGA group; this difference was statistically significant between 0 and 6 months of age. It is worth noting that there were no statistically significant differences in the prevalence of wasting among the four groups.

## 4 Discussion

Risk factors such as preterm birth, SGA, and twin births remain significant causes of neonatal death, contributing to 55% of global neonatal deaths in 2020 [13]. Considering the complex intrauterine growth environment of twins and the higher risk of gestational complications, twin SGA infants are more likely to show deviations in physical growth trajectories compared with singleton SGA [10]. A retrospective study of the data of twins treated in our hospital from 2010 to 2019 demonstrated that the risk of SGA in twin pregnancies was as high as 22.2% [4], prompting this prospective cohort study. Main findings are discussed below.

### 4.1 Within the First 6 Months of Age, Singleton SGA and Twin SGA Infants Exhibited the Fastest CUG, With Increases in Weight and Head Circumference Occurring Faster Than in Length

The concept of CUG refers to the phenomenon where SGA infants experience rapid increases in weight and height after birth. Although this compensates for intrauterine growth restriction, CUG may also increase the risk of metabolic syndrome, insulin resistance, cardiovascular disease, and other associated health issues [14]. Therefore, the establishment of moderate CUG represents a significant therapeutic strategy for SGA health management. Our findings reveal that both singleton and twin SGA infants showed the fastest CUG in weight and head circumference within 6 months, with length requiring longer to catch up, aligned with prior studies. Specifically, head circumference growth is brain development‐driven and peaks within 1–2 months [15]. Additionally, the CUG of weight started relatively early and peaked within 3 months after birth [16]. Existing studies have demonstrated that breastfeeding has a positive effect on weight and head circumference of SGA infants; however, its impact on height was not significant [10]. The mechanism underlying CUG in height is more complex than that of weight and head circumference. Growth hormone (GH) and its downstream insulin‐like growth factor‐1 (IGF‐1) are major factors that promote linear growth. Studies have shown that SGA children may exhibit low IGF‐1 levels, leading to dysfunction of the GH‐IGF‐1 axis, which affects the rate of CUG [17].

The 2023 SGA guidelines recommended monitoring growth trajectories to promote moderate CUG, as excessive weight gain may increase metabolic risks. Consequently, the current WHO policy recommends exclusive breastfeeding or standard infant formula for full‐term SGA infants, rather than high‐energy formula, to reduce the risk of overnutrition [18]. Previous studies conducted by our research group found that there was no statistically significant difference in daily energy or protein intake. This suggested that CUG in this period may not be influenced by energy intake [19]. In conclusion, given the variations in the CUG across different physical indicators in SGA infants, it is necessary to closely monitor growth patterns, especially the proportionality of weight, length, and head circumference, to ensure balanced growth and prevent excessive weight gain.

### 4.2 Differences Exist Between Singleton and Twin SGA Infants in CUG Patterns

Across all the groups, SGA exhibited the lowest mean BW. The difference in physical growth indicators between singletons and twins at birth may be associated with intrauterine growth. Management of Dichorionic Pregnancy, issued by the Society of Obstetricians and Gynecologists of Canada (SOGC) in 2022, stated that twins grow more slowly than singletons in the last trimester (especially after 30 weeks) [20]. Although twins' birth weight was lower than that of singletons, SGA twins showed a faster trend of CUG in weight after birth. The WAZ and HCZ of twins surpassed the singleton SGA group at 1 year of age. Additionally, the twin group had a lower prevalence of underweight and wasting than those of the singleton SGA group, but a higher prevalence of stunting (19.4% vs. 15.9%). Our previous retrospective study of twins found that the prevalence of stunting at 1 year of age was higher in the twins' SGA group than in the low weight group (30.8% and 26.9%) [4]. During the first year after birth, although the physical growth indicators of SGA twins could reach the normal range, they often fell below the average level of singletons of the same age [21]. Research has indicated that the length level of SGA twins can catch up with AGA twins when they are 7–9 years old [22]. A study conducted in Jinan, China, showed that the weight growth rate of twins was significantly higher than that of singletons, whereas the length growth rate was not significantly different [23]. Even after adjusting for confounding factors, the difference in weight gain between twin and singleton SGA infants remained statistically significant.

Based on existing research, the differences in CUG patterns between SGA singletons and twins are caused by various factors, including both prenatal and postnatal factors. In singleton pregnancies, the occurrence of SGA is mainly influenced by maternal, fetal, placental, and umbilical cord factors. In contrast, in twin pregnancies, the pregnancy itself constitutes a significant risk factor for SGA. In twin or multiple pregnancies, limited intrauterine space, smaller placental surface area, and relatively insufficient maternal nutritional supply can restrict normal fetal development [24]. Additionally, selective intrauterine growth restriction (sIUGR) and twin–twin transfusion syndrome (TTTS) are common complications in twin pregnancies, often associated with the occurrence of SGA in both singletons and twins. Twin SGA infants potentially experience greater health challenges and complications due to the special growth environment and the inherent competition [25]. Furthermore, the slower growth of length among twins may be related to gestational age at birth. In this study, the infants in the twin SGA group were primarily full‐term and late preterm. In a previous study involving Dutch twins, twins with a higher gestational age were found to have a slower growth rate in length between birth and 2 years of age [26]. In another study, the CUG rate was 92.3% by 2 years of age [27]. Two other studies involving twins with inconsistent birth weights found that CUG rates in the twin SGA group at age 4 years old were only 53.3% and 69%, respectively [28, 29]. It is worth noting that research has shown that catch‐up in height is influenced by genetic, environmental, and nutritional factors, and there is a correlation between short stature at 2 years of age and short stature in adulthood [30]. Therefore, the postnatal body length of SGA children should be managed proactively through early, regular monitoring and follow‐up, with timely interventions implemented when necessary. SGA twins exhibit a greater risk of stunting, and the time required to catch up with growth may be longer, necessitating close monitoring and clinical care.

### 4.3 Differences Between Girls and Boys in CUG Among SGA Twins

Analysis of the fitted growth curves for weight, length, and head circumference of SGA infants revealed differences in the CUG between sexes. This study found that the length of male twin SGA infants was lower than that of singleton SGA infants in the early stage, whereas female twin SGA infants demonstrated relatively greater body length than singleton females. The length of male infants reached −2SD to −1SD at 1 year, whereas that of female infants approached approximately 1SD. The weight of male twin SGA infants was slightly lower compared to that of singleton SGA infants in the early stage, whereas the weight levels of female twin SGA infants and singleton SGA infants were typically comparable in the early stage. However, postnatal weight catch‐up was more pronounced in male SGA twins than in females, with both sexes eventually exceeding the weight levels of singleton SGA infants by 6 months of age. Regarding head circumference, CUG was more marked in female than in male SGA infants. Multi‐regional research has shown that using non‐sex‐specific birth weight standards may result in a higher likelihood of females being classified as SGA [31]. Additionally, the placental vascular anastomoses in monochorionic twins may lead to unequal blood flow distribution, disproportionately affecting male fetuses due to their higher metabolic demands [32]. The higher testosterone levels in male fetuses may increase the metabolic rate and exacerbate the competition for resources [33]. Animal studies have also demonstrated that male fetuses may exhibit greater sensitivity to hypoxic and nutritional stress, with observed sex differences in placental epigenetic regulation, including DNA methylation patterns [34]. Additionally, research has established that male fetuses with intrauterine growth restriction (IUGR) are more prone to respiratory distress and hypoglycemia, and have an elevated risk of admission to the NICU [35]. Nonetheless, these findings have inconsistencies, with some studies indicating the absence of significant differences between sexes [31], suggesting that regional and population‐specific characteristics may influence our study findings.

Existing data indicate that there may be differences in the incidence and pathological characteristics of IUGR and SGA between the sexes in twin pregnancies. It is worth noting that females are highly likely to be misdiagnosed as SGA, with males being susceptible to severe complications. Future research should focus on conducting large‐scale, multi‐center studies to standardize diagnostic criteria and investigate sex‐specific growth patterns and intervention strategies.

### 4.4 Twin AGA Infants Exhibit Rapid CUG, Necessitating Close Monitoring to Avoid Excessive Weight Gain and Obesity

The BW of the twin AGA group was lower than that of the singleton AGA group, with rapid growth experienced by the twin infants after birth. By the age of 1 year, their growth level exceeded that of the singleton AGA group. A previous retrospective study found that twin AGA children exhibited a higher CUG rate at 1 year old compared with singleton AGA: the rates were 94.9% for weight, 87.2% for length, and 93.6% for head circumference [4]. This phenomenon may be associated with IUGR, a condition closely linked to, but not synonymous with SGA. IUGR is defined as suboptimal fetal growth, typically identified through serial ultrasound assessments. Most babies with IUGR are likely to be SGA, depending on the time and severity of intrauterine injury, however, some babies with IUGR are born with AGA. In twin pregnancies, IUGR is more frequently observed during pregnancy, even when infants are classified as AGA at birth, compared to singleton pregnancies. The IUGR rate of twin AGA is higher compared to that of single AGA [36]. McLaughlin et al. found that AGA infants with a history of IUGR had lower WAZ, LAZ, and BMIZ at birth compared to the average values of normal children. However, these infants exhibited rapid CUG immediately after birth and reached normal levels by 4 months of age [37]. This rapid postnatal growth may be associated with physiological factors such as elevated insulin sensitivity, lower leptin levels, and reduced body fat percentage.

Research has shown that infants with a history of CUG are 3.66 times more likely to be overweight in adulthood than children without CUG [38]. In the study by McLaughlin et al., the body mass index (BMI) of children with AGA who experienced IUGR at 2 years of age was not statistically different from that of children with AGA who had normal intrauterine growth rates [37]. However, Stettler et al. demonstrated that overweight at age of 7 years was significantly associated with rapid weight gain at age of 1 year [39]. In our study, although no statistically significant difference in WFLZ was observed between twin and singleton AGA infants at 1 year of age, the prevalence of overweight was notably higher in twin AGA (4.5%) than in singleton AGA (1.4%) and both singleton and twin SGA (0%). Our retrospective study similarly reported an overweight prevalence of 3.8% among one‐year‐old AGA twins, with no cases of overweight observed in the SGA group [10]. Although some AGA twins may have experienced IUGR, the necessity and potential benefits of rapid postnatal CUG in this population remain controversial, particularly given that their birth weight and length levels were normal. Therefore, twin AGA infants represent a cohort warranting closer attention. In particular, close monitoring of the changes in their body shape symmetry is essential to detect early signs of overweight or obesity, and to mitigate associated short‐ and long‐term complications.

Overall, our study underscores the need for regular follow‐up monitoring of SGA infants. Studies have shown that regular growth monitoring can detect growth deviations in a timely manner, enabling timely intervention. Children who received regular growth monitoring have been shown to have a higher probability of achieving appropriate growth indicators than those who did not receive monitoring [40]. Discussions among Chinese experts have proposed that SGA infants are highly heterogeneous. Therefore, it is necessary to comprehensively analyze and manage these infants by combining different postnatal growth patterns and management priorities at different stages to improve health outcomes and enhance the quality of life [41]. Although our study did not directly compare the differences in growth between regularly and irregularly followed‐up SGA infants, during the follow‐up process of this study, detailed knowledge education, feeding guidance, as well as exercise and sleep guidance were provided. Moreover, the children in the cohort had a higher follow‐up frequency compared to other children undergoing voluntary follow‐up. The research results showed that the CUG of SGA infants in the cohort and the correction of physical indicator deviations were generally satisfactory. Therefore, we believe that regular follow‐up for SGA infants is of great importance.

## 5 Limitations

Despite efforts to reduce selection bias, limitations remain. Parental participation bias may affect cohort representativeness, and its impact cannot be fully eliminated. Future studies should optimize recruitment to improve the generalizability of the findings. Our hospital lacks an obstetrics department, hampering prenatal data collection. Because of limitations such as poor questionnaire quality and a relatively small sample size, the results of a multivariable logistic regression analysis based on our existing data were not reliable enough. Therefore, we did not present them in the article. It is worth noting that the number of covariates included in the GEE model was limited, which may have allowed unaccounted confounding factors to affect the results. Thus, our results should be interpreted with caution. We aim to collaborate with hospitals having obstetrics departments to gather more accurate data for SGA research. Given that CUG in SGA infants often extends beyond the first year of life, we plan to conduct a longitudinal cohort study with extended follow‐up to further investigate the differences in growth patterns and underlying mechanisms of CUG.

## 6 Conclusions

The length, weight, and head circumference of SGA infants typically exhibited a CUG. By the age of 1 year, although their average growth metrics remained below those of AGA infants, the growth deviation rate had decreased significantly. It is worth noting that the CUG in height required a longer period compared to weight, suggesting that excessive concern over relatively low early postnatal weight may lead to inappropriate interventions with potential long‐term risks. There were differences in CUG between singleton and twin SGA infants, with potential sex‐specific variation among SGA twin infants. Additionally, the body proportions of twin AGA infants require close monitoring. Regular monitor of SGA is recommended. Moving forward, the development of locally tailored SGA diagnostic criteria that distinguish between fetal sex and plurality may enhance the accuracy of growth assessments and related research.

## Author Contributions

Xuan Zhang conceptualized and designed the study, acquired funding, reviewed, and revised the manuscript. Yannan Wan and Ting Pan conducted the data analysis, drafted the initial manuscript, and revised it. Yannan Wan, Zhanzhan Zhang, Yuanfeng Zhong, Qian Chen, Lin Zhu, Xuelin Xia, Li Chen, and Xuan Zhang acquired the data and were responsible for the follow‐up of these children. All authors approved the final version of this manuscript and agreed to its publication.

## Ethics Statement

The Ethic Committee of the Children's Hospital of Chongqing Medical University approved the research protocol (ethics approval number: 2021, No. 270).

## Conflicts of Interest

The authors declare that the research was conducted in the absence of any commercial or financial relationships that could be construed as a potential conflict of interest.

## Supporting information


Supporting Information S1

## Data Availability

The datasets generated and analyzed during the current study are not publicly available due to patients' privacy concerns but are available from the corresponding author upon reasonable request by contacting via e‐mail: jasminewyn1108@163.com.

## References

[1] Y. Liu , X. B. Tang , G. G. Huang , Y. Zhang , and Y. G. Li , “Analysis of the Follow‐Up Situation of 1268 High‐Risk Infants” [in Chinese], Maternal Child Health Care of China 28, no. 16 (2013): 2571–2573, 10.7620/zgfybj.j.issn.1001-4411.2013.28.26.

[2] N. Li , H. An , M. Jin , et al., “Association of Infants Small for Gestational Age With Anemia Under Five Years Old in Two Large Longitudinal Chinese Birth Cohorts,” Nutrients 14, no. 5 (2022): 1006, 10.3390/nu14051006. 35267982 PMC8912436

[3] Q. H. Wang , Y. J. Yang , K. L. Wei , and L. Z. Du , “Current Situation Investigation and Analysis of SGA in China” [in Chinese], Chinese Journal of Practical Pediatrics 24, no. 3 (2009): 177–180, https://www.cnki.com.cn/Article/CJFDTotal‐ZSEK200903010.htm.

[4] Y. R. Huang and X. Zhang , “Research Progress on the Etiology and Growth and Development Status of Twin Small for Gestational Age Infants” [in Chinese], Advances in Clinical Medicine 11, no. 5 (2021): 2139–2146, 10.12677/ACM.2021.115306.

[5] L. M. Sun , T. Duan , and H. X. Yang , “Clinical Management Guidelines for Twin Pregnancies” [in Chinese], Chinese Journal of Perinatal Diagnosis (Electronic Edition) 13, no. 1 (2021): 51–63: (Updated in 2020) 10.13470/j.cnki.cjpd.2021.01.011.

[6] E. Yalın İmamoğlu , M. Hayran , K. S. Mahir , et al., “Birth Weight Reference Percentiles by Gestational Age for Turkish Twin Neonates,” Turkish Archives of Pediatrics 56, no. 4 (2021): 316–321, 10.5152/TurkArchPediatr.2021.20259. 35005724 PMC8655957

[7] V. Giorgione , C. Briffa , C. Di Fabrizio , R. Bhate , and A. Khalil , “Perinatal Outcomes of Small for Gestational Age in Twin Pregnancies: Twin vs. Singleton Charts,” Journal of Clinical Medicine 10, no. 4 (2021): 643, 10.3390/jcm10040643. 33567545 PMC7916041

[8] X. H. Liu , “Nutritional Support and Health Management of High‐Risk Infants” [in Chinese], Chinese Journal of Child Health Care 30, no. 2 (2022): 120–123, 10.11852/zgetbjzz2022-0058.

[9] H. H. Wu , X. N. Zong , H. Li , et al., “Growth Reference Standards for the Weight/Head Circumference Ratio and Length/Head Circumference Ratio of Chinese Newborns” [in Chinese], Chinese Journal of Evidence‐Based Pediatrics 15, no. 6 (2020): 401–405, 10.3969/j.issn.1673-5501.2020.06.001.

[10] Z. X. Chen , R. Li , L. T. Peng , and X. N. Li , “Clinical Characteristics of Catch‐Up Growth in Small for Gestational Age Infants” [in Chinese], Chinese Journal of Child Health Care 32, no. 9 (2024): 1024–1028, 10.11852/zgetbjzz2023-0796.

[11] N. Nurani , T. Wibowo , R. Susilowati , J. Hastuti , M. Julia , and M. M. Van Weissenbruch , “Growth of Exclusively Breastfed Small for Gestational Age Term Infants in the First Six Months of Life: A Prospective Cohort Study,” BMC Pediatrics 22, no. 1 (2022): 73, 10.1186/s12887-021-03080-6. 35105325 PMC8805422

[12] A. Tian , F. Meng , S. Li , Y. Wu , C. Zhang , and X. Luo , “Inadequate Linear Catch‐Up Growth in Children Born Small for Gestational Age: Influencing Factors and Underlying Mechanisms,” Reviews in Endocrine & Metabolic Disorders 25, no. 4 (2024): 805–816, 10.1007/s11154-024-09885-x. 38763958 PMC11294269

[13] World Health Organization , World Health Statistics 2020: Monitoring Health for the SDGs, Sustainable Development Goals (Licence: CC BY‐NC‐SA 3.0 IGO, 2020), https://www.who.int/publications/i/item/9789240005105.

[14] P. Li‐li , S. Zhe , and Y. Jing‐yu , “Research Progress in Small for Gestational Age” [in Chinese], Chinese Journal of Practical Pediatrics 36, no. 8 (2021): 602–607, 10.19538/j.ek2021080609.

[15] C. Morgan , S. Parry , J. Park , and M. Tan , “Neurodevelopmental Outcome in Very Preterm Infants Randomised to Receive Two Different Standardised, Concentrated Parenteral Nutrition Regimens,” Nutrients 15, no. 22 (2023): 4741, 10.3390/nu15224741. 38004135 PMC10674254

[16] Y. Zhao , X. Fan , J. Wen , W. Gan , and G. Xiao , “Analysis of Longitudinal Follow‐Up Data of Physical Growth in Singleton Full‐Term Small for Gestational Age Infants,” Journal of International Medical Research 49, no. 12 (2021): 03000605211060672, 10.1177/03000605211060672. 34855533 PMC8647279

[17] J. S. Renes , J. van Doorn , and A. C. S. Hokken‐Koelega , “Current Insights Into the Role of the Growth Hormone‐Insulin‐Like Growth Factor System in Short Children Born Small for Gestational Age,” Hormone Research in Paediatrícs 92, no. 1 (2019): 15–27, 10.1159/000502739. 31509834 PMC6979433

[18] A. C. S. Hokken‐Koelega , M. van der Steen , M. C. S. Boguszewski , et al., “International Consensus Guideline on Small for Gestational Age: Etiology and Management From Infancy to Early Adulthood,” Endocrine Reviews 44, no. 3 (2023): 539–565, 10.1210/endrev/bnad002. 36635911 PMC10166266

[19] Q. Yang , Q. Cheng , and X. Zhang , “Relationship Between Energy Intake and Catch‐Up Growth Within 6 Months of Age in Full‐Term Small for Gestational Age Infants” [in Chinese], Chinese Journal of Pediatrics 53, no. 12 (2015): 919–924, 10.3760/cma.j.issn.0578-1310.2015.12.011. 26887547

[20] E. Mei‐Dan , V. Jain , N. Melamed , et al., “Guideline No. 428: Management of Dichorionic Twin Pregnancies,” Journal of Obstetrics and Gynaecology Canada 44, no. 7 (2022): 819–834.e811, https://www.sciencedirect.com/science/article/abs/pii/S1701216322003516. 35798461 10.1016/j.jogc.2022.05.002

[21] T. Pan , Y. Huang , Q. Cheng , et al., “A Retrospective Study on the Physical Growth of Twins in the First Year After Birth,” Frontiers in Nutrition 10 (2023): 1168849, 10.3389/fnut.2023.1168849. 37810921 PMC10557485

[22] J. L. Gleason , E. H. Yeung , R. Sundaram , et al., “Longitudinal Child Growth Patterns in Twins and Singletons in the Upstate KIDS Cohort,” Journal of Pediatrics 263 (2023): 113720, 10.1016/j.jpeds.2023.113720. 37660974 PMC10872829

[23] L. Zhang , Y. Li , S. Liang , X. J. Liu , and F. L. Kang , “Postnatal Length and Weight Growth Velocities According to Fenton Reference and Their Associated Perinatal Factors in Healthy Late Preterm Infants During Birth to Term‐Corrected Age: An Observational Study,” Italian Journal of Pediatrics 45, no. 1 (2019): 1, 10.1186/s13052-018-0596-4. 30606228 PMC6318852

[24] L. M. Sun , Y. Y. Zhao , T. Duan , et al., “Clinical Management Guidelines for Twin Pregnancies (Part 2)—Diagnosis and Treatment of Complications in Twin Pregnancies” [in Chinese], Chinese Journal of Perinatal Diagnosis (Electronic Edition) 7, no. 4 (2015): 57–64, 10.13470/j.cnki.cjpd.2015.04.015.

[25] Z. T. Zhang , C. X. Liu , S. W. Yin , et al., “Guidelines for the Diagnosis, Treatment and Health Care of Selective Fetal Intrauterine Growth Restriction (2020)” [in Chinese], Chinese Journal of Practical Gynecology and Obstetrics 36, no. 7 (2020): 618–625, 10.19538/j.fk2020070112.

[26] D. Jaquet , D. Collin , C. Lévy‐Marchal , and P. Czernichow , “Adult Height Distribution in Subjects Born Small for Gestational Age,” Hormone Research 62, no. 2 (2004): 92–96, 10.1159/000079709. 15263821

[27] S. Chen , Z. Liu , H. Zhu , et al., “Height at Three Months Can Indicate Overweight at Two Years in Catch‐Up Growth of Small for Gestational Age Infants,” Scientific Reports 8, no. 1 (2018): 13411, 10.1038/s41598-018-29698-8. 30194331 PMC6128850

[28] M. Grunewald , S. Schulte , P. Bartmann , et al., “Monozygotic Twins With Birth‐Weight Differences: Metabolic Health Influenced More by Genetics or by Environment?,” Hormone Research in Paediatrícs 91, no. 6 (2019): 391–399, 10.1159/000501775. 31412339

[29] S. Schulte , J. Wölfle , F. Schreiner , et al., “Birthweight Differences in Monozygotic Twins Influence Pubertal Maturation and Near Final Height,” Journal of Pediatrics 170 (2016): 288–294, 10.1016/j.jpeds.2015.12.020. 26794471

[30] S. C. Campisi , S. E. Carbone , and S. Zlotkin , “Catch‐Up Growth in Full‐Term Small for Gestational Age Infants: A Systematic Review,” Advances in Nutrition 10, no. 1 (2019): 104–111, 10.1093/advances/nmy091. 30649167 PMC6370265

[31] A. M. Inkster , I. Fernández‐Boyano , and W. P. Robinson , “Sex Differences Are Here to Stay: Relevance to Prenatal Care,” Journal of Clinical Medicine 10, no. 13 (2021): 3000, 10.3390/jcm10133000. 34279482 PMC8268816

[32] M. A. Shanahan and M. W. Bebbington , “Placental Anatomy and Function in Twin Gestations,” Obstetrics and Gynecology Clinics North America 47, no. 1 (2020): 99–116, 10.1016/j.ogc.2019.10.010. 32008674

[33] E. R. van der Vlugt , P. E. Verburg , S. Y. Leemaqz , et al., “Sex‐ and Growth‐Specific Characteristics of Small for Gestational Age Infants: A Prospective Cohort Study,” Biology of Sex Differences 11, no. 1 (2020): 25, 10.1186/s13293-020-00300-z. 32370773 PMC7201715

[34] Z. He , H. Lu , H. Luo , et al., “The Promoter Methylomes of Monochorionic Twin Placentas Reveal Intrauterine Growth Restriction‐Specific Variations in the Methylation Patterns,” Scientific Reports 6, no. 1 (2016): 20181, 10.1038/srep20181. 26830322 PMC4735741

[35] A. Marzouk , A. Filipovic‐Pierucci , O. Baud , et al., “Prenatal and Post‐Natal Cost of Small for Gestational Age Infants: A National Study,” BMC Health Services Research 17, no. 1 (2017): 221, 10.1186/s12913-017-2155-x. 28320392 PMC5359886

[36] M. L. E. Hendrix , S. M. J. van Kuijk , S. E. El Bahaey , et al., “Postnatal Growth During the First Five Years of Life in SGA and AGA Neonates With Reduced Fetal Growth,” Early Human Development 151 (2020): 105199, 10.1016/j.earlhumdev.2020.105199. 33032049

[37] E. J. McLaughlin , R. J. Hiscock , A. J. Robinson , et al., “Appropriate‐for‐Gestational‐Age Infants Who Exhibit Reduced Antenatal Growth Velocity Display Postnatal Catch‐Up Growth,” PLoS One 15, no. 9 (2020): e0238700, 10.1371/journal.pone.0238700. 32898169 PMC7478563

[38] M. Zheng , K. E. Lamb , C. Grimes , et al., “Rapid Weight Gain During Infancy and Subsequent Adiposity: A Systematic Review and Meta‐Analysis of Evidence,” Obesity Reviews 19, no. 3 (2018): 321–332, 10.1111/obr.12632. 29052309 PMC6203317

[39] N. Stettler , B. S. Zemel , S. Kumanyika , and V. A. Stallings , “Infant Weight Gain and Childhood Overweight Status in a Multicenter, Cohort Study,” Pediatrics 109, no. 2 (2002): 194–199, 10.1542/peds.109.2.194. 11826195

[40] Y. Cao , Y. Liang , X. Peng , and M. Sun , “Long‐Term Impacts of Growth and Development Monitoring: Evidence From Routine Health Examinations in Early Childhood,” in CESifo Working Paper, No. 10912 (Center for Economic Studies and ifo Institute [CESifo], 2024), https://api.semanticscholar.org/CorpusID:260085651. 10.1016/j.jhealeco.2025.102972 40239324

[41] W. Wu and X. Luo , “Lifelong Management of Small for Gestational Age Infants” [in Chinese], Journal of Chongqing Medical University 47, no. 3 (2022): 256–258, 10.13406/j.cnki.cyxb.003000.

