# Supplementary material for: Postnatal Growth Patterns and Deviations in Singleton and Twin Infants: A Prospective Cohort Study on Infants Appropriate and Small for Gestational Age
Source: Pediatr Discov. 2025 Nov 30;3(4):e70019. doi: 10.1002/pdi3.70019 (PMC12753020; doi:10.1002/pdi3.70019)
Supplement: Supplementary file 1 — Supporting Information S1 [file PDI3-3-e70019-s001.docx]

**Appendix：**

**Supplementary Materials:**

Evaluation of the effect of fitting growth curves.

| Length | Model | Logistic | | | |
| --- | --- | --- | --- | --- | --- |
|  | function | y = A2 + (A1-A2)/(1 + (x/x0)^p^) | | | |
|  | *R*²（Boys） | 0.90 | 0.90 | 0.90 | 0.88 |
|  | *R*²（Girls） | 0.93 | 0.92 | 0.91 | 0.92 |
| Weight | Model | SGompertz | | | |
|  | function | y = a×exp(-exp(-k×(x-xc))) | | | |
|  | *R*²（Boys） | 0.82 | 0.80 | 0.77 | 0.80 |
|  | *R*²（Girls） | 0.75 | 0.79 | 0.78 | 0.78 |
| Head Circumstance | Model | Logistic | | | |
|  | function | y = A2 + (A1-A2)/(1 + (x/x0)^p^) | | | |
|  | *R*²（Boys） | 0.87 | 0.86 | 0.83 | 0.91 |
|  | *R*²（Girls） | 0.83 | 0.88 | 0.86 | 0.82 |

**The Meaning of *R*^2^ for the Fitted Curve**

*R*^2^, that is, the coefficient of determination, is an indicator used to measure the goodness of fit of a regression model. It represents the proportion of the total variation of the dependent variable that can be explained by the independent variables through the fitted curve. The value of *R*^2^ ranges from 0 to 1. The closer *R*^2^ is to 1, the better the fitting effect. Usually, it is considered that when *R*^2^ reaches above 0.7, the fitting effect is relatively ideal.
